# The life cycle of *Dermacentor nuttalli* from the Qinghai-Tibetan Plateau under laboratory conditions and detection of spotted fever group *Rickettsia* spp.

**DOI:** 10.3389/fvets.2023.1126266

**Published:** 2023-02-24

**Authors:** Hejia Ma, Jingkai Ai, Ming Kang, Jixu Li, Yali Sun

**Affiliations:** ^1^State Key Laboratory of Plateau Ecology and Agriculture, Qinghai University, Xining, China; ^2^College of Agriculture and Animal Husbandry, Qinghai University, Xining, China; ^3^Qinghai Provincial Key Laboratory of Pathogen Diagnosis for Animal Diseases and Green Technical Research for Prevention and Control, Qinghai University, Xining, China

**Keywords:** *Dermacentor nuttalli*, life cycle, *Rickettsia*, molecular identification, Qinghai-Tibetan Plateau

## Abstract

*Dermacentor nuttalli* has been a focus of study because tick-borne pathogens have been widely identified in this tick from northern and southwestern China. The aim of this study was to characterize the life cycle of *D. nuttalli* under laboratory conditions and to detect spotted fever group (SFG) *Rickettsia* in the midgut and salivary glands of both field-collected and first laboratory generation adults. *D. nuttalli* ticks were collected in the field on the Qinghai-Tibetan Plateau from March to April 2021 and their life cycle was studied under laboratory conditions. Tick identify was molecularly confirmed, and SFG *Rickettsia* were detected in the midgut and salivary glands of males and females by PCR targeting different rickettsial genes. The results showed that the life cycle of *D. nuttalli* under laboratory conditions was completed in an average of 86.1 days. High positivity of *Rickettsia* spp. was detected in the midgut and salivary glands of both males (92.0%) and females (93.0%) of field-collected *D. nuttalli* ticks. However, a relatively lower positivity (4.0–6.0%) was detected in first laboratory generation adults. Furthermore, sequencing analysis showed that the *Rickettsia* sequences obtained in this study shared 98.6 to 100% nucleotide identity with *Rickettsia slovaca* and *Rickettsia raoultii* isolated from *Dermacentor* spp. in China. Phylogenetic analysis of *Rickettsia* spp. based on the *gltA, ompA, ompB* and *sca4* genes revealed that the *Rickettsia* sequences obtained could be classified as belonging to *R. slovaca* and *R. raoultii* clades. This study described for the first time the life cycle of *D. nuttalli* from the Qinghai-Tibetan Plateau under laboratory conditions. Two species of SFG *Rickettsia* were detected in the midgut and salivary glands of males and females in both field-collected and first laboratory-generation adults of *D. nuttalli*. Our study provides new insights into pathogen detection in ticks in the Qinghai-Tibet Plateau, and the relationships among hosts, ticks, and pathogens.

## Introduction

Ticks are vectors transmitting a large number of pathogenic bacteria, protozoa, and viruses to humans and animals that cause serious diseases (1, 2). Species belonging to the genus *Dermacentor* (Acari: Ixodidae), including *D. marginatus, D. nuttalli* and *D. silvarum* are well known as vectors for a great variety of infectious pathogens (3, 4). Among *Dermacentor* spp., *D. nuttalli* and *D. silvarum* are widely distributed in northern China, Mongolia, and Russia (5–9). *D. nuttalli* as a major vector for transmitting various diseases including tick-borne rickettsiosis, Q fever, anaplasmosis, and tick-borne encephalitis virus, has been reported in Europe and some countries in Asia (3, 9–16). In China, it was highlighted because the genus *Rickettsia* has been widely identified in *D. nuttalli* in northern and southwestern regions of the country (8, 16–29).

Tick-borne rickettsiosis, which is caused by species of spotted fever group (SFG) *Rickettsia*, poses a serious threat to the health of humans and animals (30–32). The SFG *Rickettsia* species, including *R. raoultii, R. slovaca, R. sibirica, R. conorii, R. rickettsii, R. heilongjiangensis, R. aeschlimannii, R. felis, R. massilia*e, and *R. monacensis*, have been identified in ticks, humans, and animals in China (23, 25, 33–38). Among them, *R. raoultii* and *R. heilongjiangensis* were identified in tick vectors and humans, and are pathogenic factors of human rickettsiosis in China (39–41).

The salivary glands and midgut in ticks, as specific and major barriers to efficient pathogen transmission, are the main factors influencing the acquisition, maintenance, colonization, and transmission of pathogens by ticks (42). The tick's salivary glands can transmit pathogens to the host during blood-feeding along with salivary secretions, and the midgut determines pathogen colonization and survival in ticks (42–45). The influences of salivary glands and the midgut on pathogen transmission and survival is not independent, rather there is a pathogenic life cycle: the salivary glands are infected by the ingestion of the infected host blood, the pathogens colonize and multiply after they are transported into the midgut along with the blood, pathogens are exchanged between the midgut and salivary glands, and the salivary glands transmit the pathogens to another host when the ticks blood-feed again (42). Studies of the genus *Rickettsia* in both the midgut and salivary glands of *D. nuttalli* are limited. The aim of this study was to characterize the life cycle of *D. nuttalli* under laboratory conditions, and to detect SFG *Rickettsia* in the midgut and salivary glands of *D. nuttalli* males and females in both field-collected and first laboratory-generation adults.

## Materials and methods

### Sample collection of ticks

**Figure 1:**
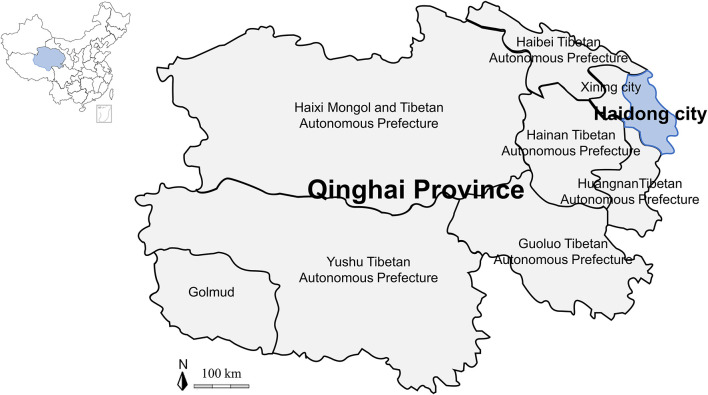
Map of the Qinghai-Tibetan Plateau area and Qinghai Province showing the sampling area in this study.

Adult ticks were collected on vegetation from 8 counties in Haidong city (102°09′-102°47′ E and 36°16′-36°40′ N, average elevation of more than 2,500 m above sea level) of Qinghai Province (89°35–103°04′ E and 31°36′-39°19′N) on the Qinghai-Tibetan Plateau (73°19′-104°47′ E and 26°00′-39°47′ N) in northwestern China from March to April 2021 (Figure 1). All ticks were placed in sterile glass bottles, sent to Qinghai University within one day, and reared in an incubator at 25–30°C and 60–90% humidity.

### Morphological identification of tick species and laboratory-rearing of ticks

Microscopy identification of tick species and sex was performed at Qinghai University. After morphological identification (46), 200 males and 200 females of *D. nuttalli* were selected for laboratory rearing.

To characterize the life-cycle of *D. nuttalli*, New Zealand white rabbits (specific-pathogen-free, female, 9-weeks-old, purchased from Yifengda, Xi'an, China) housed in the animal facility of Qinghai University at a temperature of 25 ± 2°C and humidity of 40% under controlled lighting (i.e., period of light from 6:00 to 19:00 h) were used to feed the adult ticks. After a few days, engorged ticks were weighed, placed in a sterile glass bottle, and reared in an incubator at 25 ± 2°C and 80% humidity. After tick eggs were laid and hatched, a total of 500 larvae were allowed to refeed on the ears of rabbits within 3–4 days after hatching all eggs. The engorged ticks were collected for incubation until molting as nymphs and then allowed to feed on the rabbit ears again within 12–14 days after molting all larvae. During these periods, the processes of egg laying, hatching, and molting and the viability of the ticks were regularly observed. All animal procedures were carried out according to the ethical guidelines of Qinghai University (2020017 and SL-2022007).

### DNA extraction and molecular characterization of ticks

In this study, after morphological identification of the field-collected *D. nuttalli*, we randomly selected five females and males to extract DNA for molecular identification of ticks. And for the first-laboratory generation, we also randomly selected five males and five females for molecular identification. DNA from the field-collected and first-laboratory generation adult males and females was extracted using the QIAamp DNA Mini Kit (QIAGEN, Germany) according to the manufacturer's manual. The DNA concentration was determined using a NanoDrop 2000 (Thermo Fisher Scientific, USA), and the DNA was stored at−80°C until further use. To further confirm the tick species, PCRs based on the 12S ribosomal RNA (12S rRNA), 16S ribosomal RNA (16S rRNA), cytochrome c oxidase subunit I (*COI*), and internal transcribed spacer 2 (*ITS2*) genes were conducted, respectively. All primers are listed in Table 1. The PCR assay volume was 10 μl including 2 μl of DNA, 0.5 μl of each forward and reverse primer (100 μM), 0.2 μl of deoxyribonucleotide triphosphate (200 μM, New England BioLab, USA), 1 μl of 10× ThermoPol Reaction Buffer (New England BioLab, USA), 0.1 μl of Taq polymerase (0.5 U, New England BioLab, USA) and enough double-distilled water to reach a final volume of 10 μl. Double-distilled water was used as a negative control and *D. nuttalli* DNA stored in our laboratory was used as a positive control.

**Table 1:** Oligonucleotide primers used in this study.

**Organism**	**Target gene**	**Primer sequence (5^′^-3^′^)**	**Amplification size (bp)**	**Annealing temperature(°C)**	**Reference**
*Dermacentor*. spp	12S rRNA	AAACTAGGATTAGATACCCT	386	51	(46)
		AATGAGAGCGACGGGCGATGT
	16S rRNA	ATGAAAATCTTTAAATTGCTG	475	50	(29)
		CCTCATTCTC ATCGGTCT
	*COI*	CGAATAGAACTTAGCCAACCT	1,210	50	(29)
		AATAACGACGGGGTATTCCT
	*ITS2*	TCCGTCGACTCGTTTTGACC	1,042	57	(29)
		GGATAC ATCGCTTTCGCCCAT
*Rickettsia*. spp	*ompA*	GCTTTATTCACCACCTCAAC	209/212	55	(47)
		TRATCACCACCGTAAGTAAAT
	*ompB*	AAACAATAATCAAGGTACTGT	812	53	(48)
		TACTTCCGGTTACAGCAAAGT
	*gltA*	GCAAGTATCGGTGAGGATGTAAT	401	48	(49)
		GCTTCCTTAAAATTCAATAAATCAGGAT
	*sca4*	CGATGGTAGCATTAAAAGCT	623	55	(50)
		CTTGCTTTTCAGCAATATCAC

### Detection and identification of SFG *Rickettsia*

To identify SFG *Rickettsia* in the midgut and salivary glands in both field-collected and first-laboratory generation males and females of *D. nuttalli* in the Qinghai-Tibetan Plateau area, organ DNA from 100 original males, 100 original females, 100 first-laboratory generation males and 116 first-laboratory generation females chosen randomly was extracted and screened using previously established PCR assays based on the citrate synthase gene (*gltA*). To further confirm SFG *Rickettsia* species, all samples positive for the *gltA* gene were tested for the outer membrane protein A (*ompA*) gene, outer membrane protein B (*ompB*) gene, and surface cell antigen 4 (*sca4*) gene. All primers for the four genes are listed in Table 1. The PCRs were performed as described above.

### Sequence analysis

For the confirmation of ticks and detected pathogens, at least one positive sample of each tissue per sex was selected for sequencing and molecular characterization. PCR products of the positive samples were purified using a QIAquick Gel Extraction Kit (QIAGEN, Germany). Purified PCR products was cloned into *E. coli* DH5α using the PMDTM 19-T Vector Cloning Kit (TaKaRa, Japan). At least three positive clones were sequenced by Sangon Biotech (Shanghai) Co., Ltd. The obtained sequences were confirmed by a BLASTn search in GenBank.

### Phylogenetic analyses

Phylogenetic trees were constructed using the maximum likelihood statistical method and bootstrap analysis with 500 replications in MEGA7: Molecular Evolutionary Genetics Analysis version 7.0 for larger datasets (51).

### Statistical analysis

The chi-squared test was used to compare proportions of detected sample positivity in different regions and among different animals *via* Prism 8 software. Observed differences were considered to be statistically significant when the resulting *P*-values were <0.05.

## Results

### Morphological identification and molecular characterization of *D. nuttalli*

**Figure 2:**
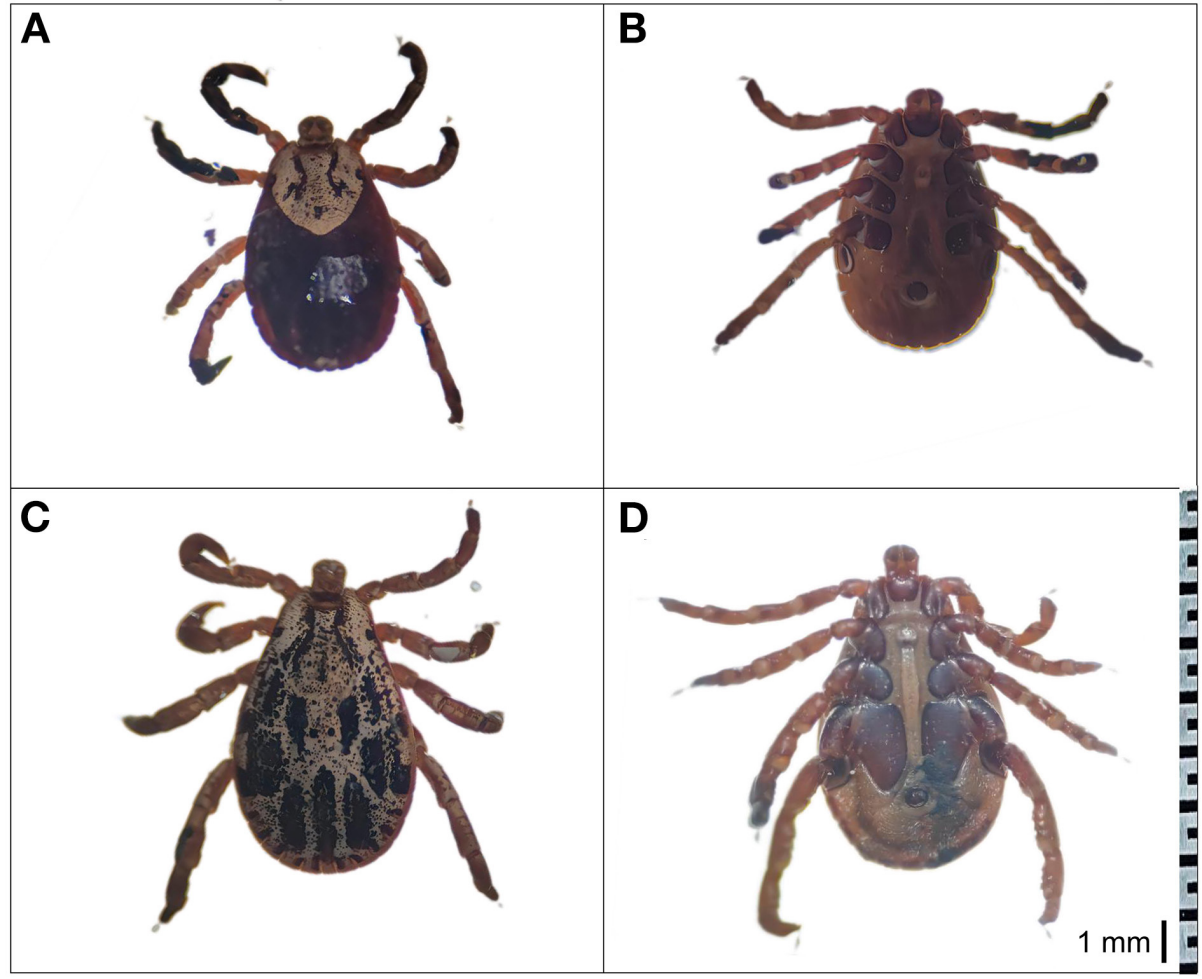
Morphological characterization of *D. nuttalli*. The dorsal view and ventral view of *D. nuttalli*. All figures used the same ruler (1 mm). **(A)** Dorsal view of female. **(B)** Ventral view of female. **(C)** Dorsal view of male. **(D)** Ventral view of male.

In this study, *D. nuttalli* ticks were preliminarily morphologically identified. As shown in Figure 2, the dorsal view of the female *D. nuttalli* is about 5.1 × 3.0 mm with an elliptical scutum of the specimen, which only occupies a small part of the front of the body (Figure 2A). And the genital pore of the female has a wing-like protrusion (Figure 2B). The male *D. nuttalli* is about 5.8 × 4.0 mm. Its scutum on the back almost covers the entire back of the body and has a lighter enamel color on its back (Figure 2C). In the ventral view of male *D. nuttalli*, the paraproct is very obvious (Figure 2D). Moreover, the capitulum of the male and female *D. nuttalli* is rectangular and the overall color is dark brown. The anal groove presents an inverted “U” shape in front of the anus.

**Figure 3:**
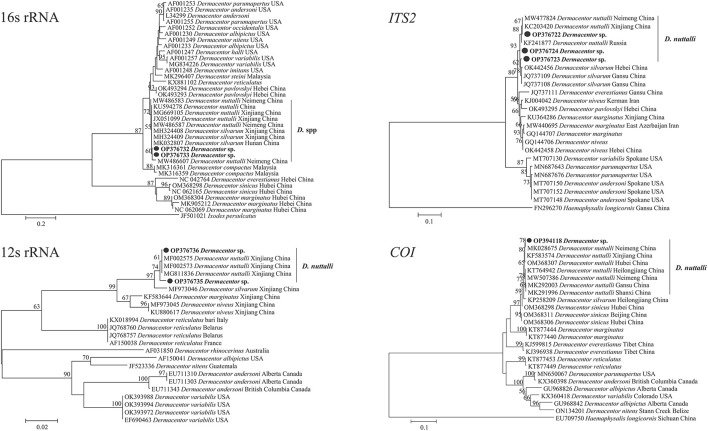
Molecular phylogenetic analysis by the maximum likelihood method based on the 12S rRNA, 16S rRNA, *COI*, and *ITS2* genes of *D. nuttalli*. The sequences obtained in this study are shown in bold font. The numbers at the nodes represent bootstrap support values.

Furthermore, the species identify was confirmed by molecular analysis of the PCR assay based on the 12S rRNA, 16S rRNA, *COI*, and *ITS2* genes. The sequencing of positive samples showed that the length of the obtained tick sequences varied from 386 to 1,210 bp (Table 2). The BLASTn analysis of two *D. nuttalli* 12S rRNA sequences in this study showed identities ranging from 98.7 to 99.5% with reference sequences in China from GenBank (Table 2). The 16S rRNA sequences of *D. nuttalli* obtained in this study showed 99.8% identity with each other and 98.2–99.0% identity with the sequences from *Dermacentor* spp. in China. Sequence analysis revealed that the nucleotide sequence of *COI* in this study shared 99.0 to 99.7% identity with *Dermacentor* spp. sequences from China and 99.0–99.7% identity with *Dermacentor* spp. sequences from other countries from GenBank. Moreover, the BLASTn analysis of the *ITS2* gene revealed that the three sequences obtained in this study had 98.7 to 99.2% identity with each other, and 97.5–99.2% identity with the sequences of *Dermacentor* spp. from China. Additionally, the phylogenetic analysis of *Dermacentor* spp. based on the 12S rRNA, 16S rRNA, *COI*, and *ITS2* genes revealed that tick sequences obtained from this study grouped with the *D. nuttalli* clades (Figure 3).

**Table 2:** *Dermacentor* spp. sequences obtained in this study.

**Obtained sequences**				**The closest BLASTn match**		
**Organism**	**Target gene**	**Accession number**	**Length (bp)**	**Identity (%)**	**Species**	**Accession number (host, country)**
*Dermacentor* spp.	12S rRNA	OP376735	386	99.2	*D. nuttalli*	MF002575.1 (*Dermacentor nuttalli*, China)
		OP376736	386	99.5	*D. nuttalli*	MF002575.1 (*Dermacentor nuttalli*, China)
	16S rRNA	OP376732	475	98.3	*D. nuttalli*	KT764942.1 (*Dermacentor nuttalli*, China)
		OP376733	475	98.5	*D. nuttalli*	KT764942.1 (*Dermacentor nuttalli*, China)
	*COI*	OP394118	1.210	99.5	*D. nuttalli*	OM368307.1 (*Dermacentor nuttalli*, China)
	*ITS2*	OP376722	1,042	99.6	*D. nuttalli*	MK027319.1 (*Dermacentor nuttalli*, China)
		OP376723	1,039	98.5	*D. nuttalli*	MW477811.1 (*Dermacentor nuttalli*, China)
		OP376724	1,045	90.0	*D. nuttalli*	KF241877.1 (*Dermacentor nuttalli*, Russia)

### Laboratory-rearing of *D. nuttalli*

**Figure 4:**
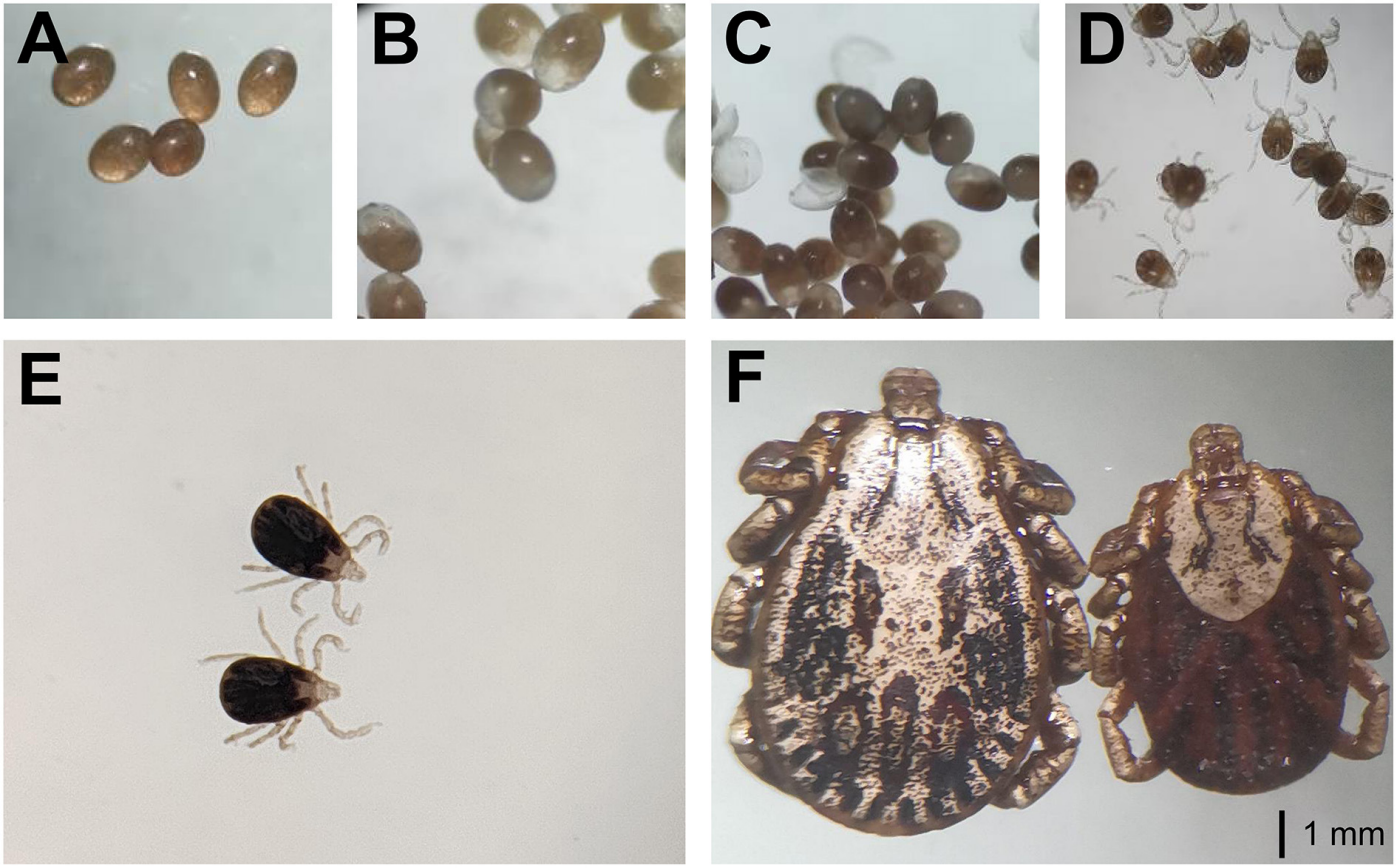
The laboratory-rearing of *D. nuttalli*. All figures used the same ruler (1 mm). **(A)** Prophase of eggs. **(B)** Metaphase of eggs. **(C)** Anaphase of eggs. **(D)** Larva. **(E)** Nymphs. **(F)** Female and male adults.

For laboratory-rearing of *D. nuttalli* and detection of *Rickettsia* colonization in first-laboratory generation *D. nuttalli*, 200 *D. nuttalli* males and 200 females were randomly chosen to generate next-generation adults by blood-biting in rabbits and rearing in an incubator at 25 ± 2°C and 80% humidity. The results showed that the first laboratory-generation of adults was completed within an average of 86.1 days (Figure 4; Table 3). The average weight of engorged females was 656.0 mg, which was 74.5 times the weight of unfed females (Table 4). No significant difference in average weight was found between field-collected and first-laboratory generation female adults, while the first-generation males produced in the laboratory weighed 2 mg more than the original male adults (Table 4).

**Table 3:** The durations of various developmental stages of *D. nuttalli*.

**Developmental stage**	**Period**	**Number of ticks tested**	**Duration (days)**	
			**Range**	**Mean ±SD**
Egg	Incubation	100	5–14	8.40 ± 2.76
Larva	Pre-feeding	100	3–4	3.71 ± 0.45
	Feeding	100	4–7	5.40 ± 0.99
	Pre-molting	100	7–8	7.29 ± 0.45
Nymph	Pre-feeding	100	5–6	5.43 ± 0.86
	Feeding	100	6–10	8.14 ± 1.25
	Pre-molting	100	5–8	7.40 ± 0.66
Adult female	Pre-feeding	25	8–11	10.16 ± 0.88
	Feeding	25	11–12	11.36 ± 0.48
	Pre-oviposition	25	2–9	4.96 ± 2.41
	Oviposition	25	9–18	13.80 ± 2.07

SD, standard deviation.

**Table 4:** Changes of body weight of larva, nymph, and adult *D. nuttalli* before and after feeding.

**Developmental stage**	**Number of ticks**	**Unfed (mg) (Mean ±SD)**	**Engorged (mg) (Mean ±SD)**	**Weight ratio (engorge/unfed)**
**Original**				
Adult male	25	7.48 ± 0.37	8.12 ± 0.41	1.09
Adult female	25	8.80 ± 0.62	656.00 ± 29.77	74.50
**First generation under laboratory conditions**				
Larva	100	0.05 ± 0.00	0.55 ± 0.07	11.00
Nymph	100	0.38 ± 0.05	24.83 ± 2.39	65.34
Adult male	25	9.48 ± 0.45	10.05 ± 0.66	1.06
Adult female	25	8.68 ± 0.48	283.28 ± 89.79	31.97

SD, standard deviation.

### Identification of SFG *Rickettsia* in the midgut and salivary glands in field-collected and first-laboratory generation *D. nuttalli* males and females

In this study, 100 field-collected *D. nuttalli* males and 100 females collected were chosen randomly to determine *Rickettsia* colonization. For first laboratory-generation ticks, 100 males and 116 females were screened by randomly selecting one or two males or females for each female mother tick. SFG *Rickettsia* in the midgut and salivary glands of the adult *D. nuttalli* males and females was determined using a PCR assay based on the *gltA* gene. As shown in Table 5, high colonization rates of *Rickettsia* were found in both the midgut and the salivary glands of both field-collected *D. nuttalli* males and females, and no positivity was found in single tissue colonization. However, low rates (4.0–6.0%) of *Rickettsia* colonization in the midgut or salivary glands of first-laboratory generation *D. nuttalli* were detected (Table 5). The positivity rates of SFG *Rickettsia* in the same generation of ticks showed no significant difference depending on either sex and organ.

**Table 5:** Infection rate (%) of SFG *Rickettsia* spp. in field-collected and first-laboratory generation adults of *D. nuttalli* in the QTPA.

***Rickettsia***	**Infection rate (%, 95% CI) in field-collected and first-laboratory generation of** ***D. nuttalli***											
	**Field-collected**						**First-laboratory generation**					
	**Male (*****n*** = **100)**			**Female (*****n*** = **100)**			**Male (*****n*** = **100)**			**Female (*****n*** = **116)**		
	**Single SG**	**Single MG**	**SG** + **MG**	**Single SG**	**Single MG**	**SG** + **MG**	**Single SG**	**Single MG**	**SG** + **MG**	**Single SG**	**Single MG**	**SG** + **MG**
Positive	0	0	92 (92.0, 86.7–97.3)	0	0	93 (93.0, 88.0–98.0)	4 (4.0, 0.2–7.8)	6 (6.0, 1.3–10.7)	0	5 (4.3, 0.6–8.0)	5 (4.3, 0.6–8.0)	0
Negative	/	/	8 (8.0, 2.7–13.3)	/	/	7 (7.0, 2.0–12.0)	96 (96.0, 92.2–99.8)	94 (94.0, 89.3–98.7)	/	111 (95.7, 92.0–99.4)	111 (95.7, 92.0–99.4)	/

SG, salivary gland. MG, midgut. 95% CI, 95% confidence interval. /, no analysis.

**Figure 5:**
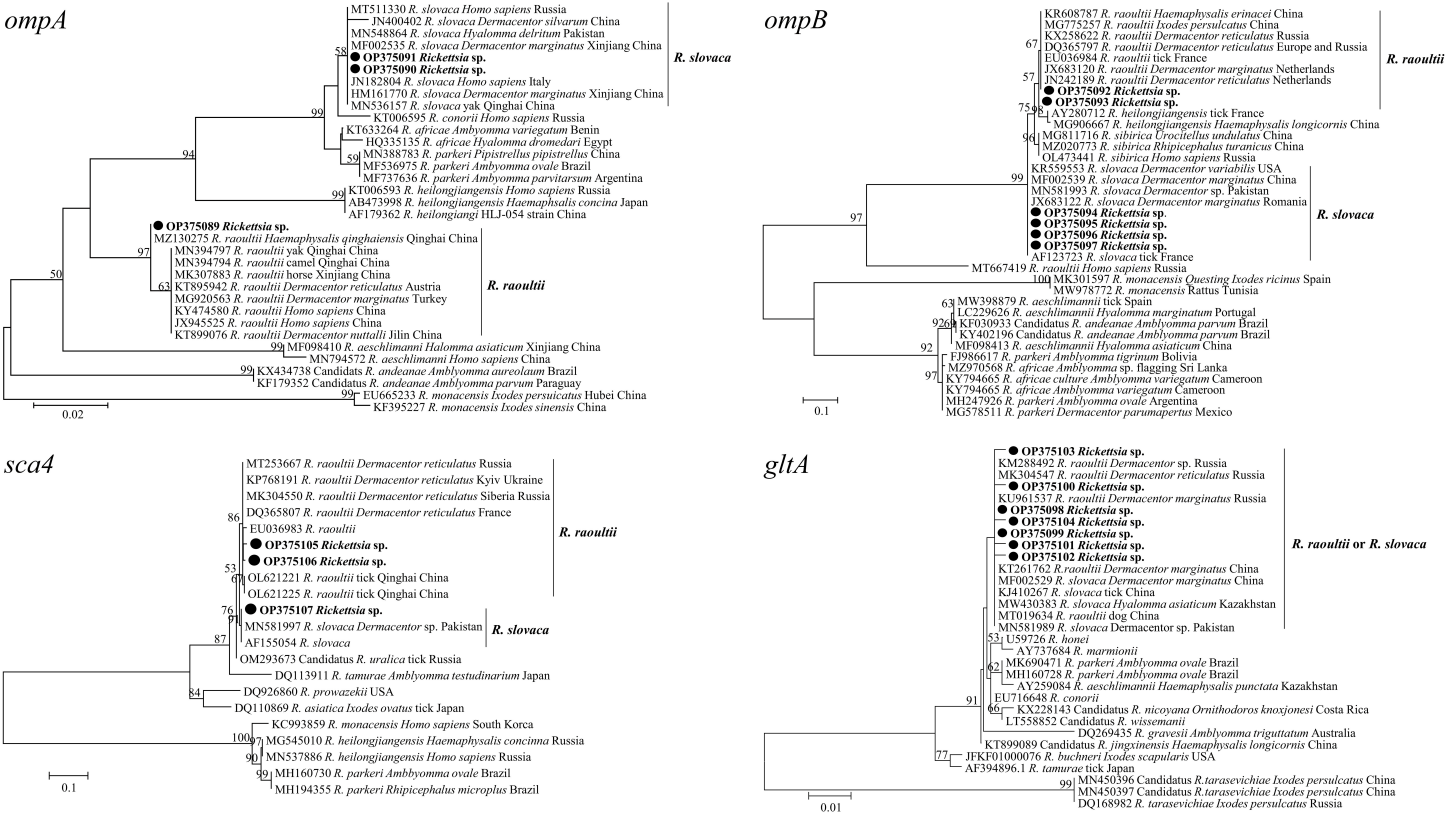
Molecular Phylogenetic analysis by maximum likelihood method based on the *gltA, ompA, ompB*, and *sca4* genes of spotted fever group *Rickettsia*. The sequences obtained in this study are shown in bold font. The numbers at the nodes represent bootstrap support values.

All positive samples for the *gltA* gene were confirmed by PCR of the *ompA, ompB* and *sca4* genes, and the GenBank numbers of these sequences with lengths ranging from 209 to 813 bp are listed in Table 6. BLASTn analysis of the four genes showed that the *Rickettsia* spp. detected in this study had 98.6 to 100% identities with either *R. slovaca* or *R. raoultii* sequences from *Dermacentor* spp. from China. The phylogenetic analysis of *Rickettsia* sequences based on the *gltA, ompA, ompB* and *sca4* genes revealed that the sequences obtained from *D. nuttalli* in this study grouped with the *R. slovaca* and *R. raoultii* clades of isolates from *Dermacentor* spp., *Homo sapiens*, animals and other tick species from China, Russia, France, Pakistan, the United States, the Netherlands, Turkey, Romania, Austria, and Italy (Figure 5).

**Table 6:** *Rickettsia* spp. sequences obtained in this study.

**Obtained sequences**				**The closest BLASTn match**		
**Organism**	**Target gene**	**Accession number**	**Length (bp)**	**Identity (%)**	**Species**	**Accession number (host, country)**
*Rickettsia*	*ompA*	OP375089	209	100	*R. raoultii*	MN394801.1 (*yak*, China)
		OP375090	212	100	*R. slovaca*	MN394806.1 (*yak*, China)
		OP375091	212	100	*R. slovaca*	MN388785.1 (*Pipistrellus pipistrellus*, China)
	*gltA*	OP375098	401	100	*R. raoultii*	MK304547.1 (*Dermacentor reticulatus*, Russia)
		OP375099	401	100	*R. slovaca*	MK304547.1 (*Dermacentor reticulatus*, Russia)
		OP375100	401	99.8	*R. slovaca*	MK304547.1 (*Dermacentor reticulatus*, Russia)
		OP375101	401	99.8	*R. slovaca*	MK304547.1 (*Dermacentor reticulatus*, Russia)
		OP375102	401	99.8	*R. slovaca*	MK304547.1 (*Dermacentor reticulatus*, Russia)
		OP375103	401	99.8	*R. slovaca*	MF002529.1 (*Dermacentor marginatus*, China)
		OP375104	401	99.8	*R. slovaca*	MF002529.1 (*Dermacentor marginatus*, China)
	*ompB*	OP375092	812	99.9	*R. raoultii*	MF002526.1 (*Dermacentor nuttalli*, China)
		OP375093	812	99.8	*R. raoultii*	MF002526.1 (*Dermacentor nuttalli*, China)
		OP375094	812	99.9	*R. slovaca*	MF002539.1 (*Dermacentor marginatus*, China)
		OP375095	813	99.8	*R. slovaca*	MF002539.1 (*Dermacentor marginatus*, China)
		OP375096	812	99.9	*R. slovaca*	MF002539.1 (*Dermacentor marginatus*, China)
		OP375097	812	100	*R. slovaca*	MF002539.1 (*Dermacentor marginatus*, China)
	*sca4*	OP375105	624	99.0	*R. raoultii*	KP768191.1 (*Dermacentor reticulatus*, Ukraine)
		OP375106	624	99.8	*R. raoultii*	CP098324.1 (*Dermacentor silvarum*, China)
		OP375107	624	100	*R. slovaca*	MN581997.1 (*Dermacentor* sp., Pakistan)

## Discussion

The current sampling sites located in Qinghai Province on the Qinghai-Tibetan Plateau are over 2,500 m above sea level and have a unique natural ecosystem with a cold climate and oxygen deficiency. Some organisms, such as the yak (*Bos grunniens*) and tick vectors, can survive under the extreme environmental conditions of Qinghai Province. Chen et al. (52) reported that at least 25 species of six tick genera have been recorded in Qinghai Province, and the most common species were *Haemaphysalis qinghaiensis, D. nuttalli* and *D. silvarum* (24). In this study, the collected *D. nuttalli* ticks in this province were molecularly confirmed, and found to have a life cycle with an average of 86.1 days under laboratory conditions. Furthermore, the present study detected two species of SFG *Rickettsia* that grouped with *R. slovaca* and *R. raoultii* in both the salivary glands and midgut of field-collected and first-laboratory generation males and females.

The life-cycle of several tick species including *D. everestianus, Ha. qinghaiensis, Hyalomma scupense, Hy. asiaticum* and *Hy. rufipes* under laboratory conditions and *D. silvarum, Ha. tibetensis* and *Ha. concinna* under field conditions, has been characterized in China (53–60). These studies showed that the average period of one female tick life-cycle of under laboratory conditions was ranged from 110.2 to 179.0 days, while it ranged from 121.0 to 177.8 days under field conditions (53–60). However, the average periods showed seasonal differences (53–60). *D. nuttalli* is widely distributed in northern China, including the sampling sites in Qinghai Province in this study (5, 6, 8). Here, an average life cycle of 86.1 days (ranging from 65.0 to 107.0 days) of *D. nuttalli* under laboratory conditions was found, indicating a shorter length of time than previously reported (53–60). This may be because the ticks were collected in March and April which is the blood-feeding period in the natural cycle, and ticks may retain wild habits during the first cycle in the laboratory. These results suggest the specificity of the life cycle of *D. nuttalli* compared with other hard ticks, especially in terms of the duration and the morphology of different life stages.

The ubiquity and high proportions of pathogens in the salivary glands and midgut of ticks collected in the field were determined by the species of pathogens and the presence of infectious host blood. The present study detected SFG *Rickettsia* in both the salivary glands and midgut of males and females, with high infection rates in the original *D. nuttalli* adults. This suggests that this tick-borne pathogen could be well adapted to the different environmental conditions of both the salivary glands and the midgut in *D. nuttalli* males and females, which is consistent with the finding of previous studies that SFG *Rickettsia* was most frequent in *D. nuttalli* ticks in Qinghai Province, northwestern China (24). Subsequently, the identification of *Rickettsia* was performed in the first-laboratory generation adults of *D. nuttalli*. However, the result in contrast with that in the original ticks, in which SFG *Rickettsia* was detected in either the salivary glands or the midgut in males and females, with low positivity rates. This indicates that the acquisition, maintenance, colonization, and transmission of SFG *Rickettsia* by *D. nuttalli* ticks cannot be separated from the blood contributions of an effective host. Moreover, although our study simulated the natural environment of ticks, it is possible that subtle environmental changes could influence the life-cycle of ticks, as well as the colonization and exchange of pathogens in tick salivary glands or midguts (53–60). Furthermore, the positivity rates of SFG *Rickettsia* in the same generation of ticks showed no significant difference between sexes or organs, suggesting that this pathogen strongly colonizes in both the salivary glands and the midgut in *D. nuttalli* males and females.

Furthermore, our study provides new evidence of DNA-based detection of SFG *Rickettsia* pathogens in *D. nuttalli* ticks in the sampling areas. The sequencing and phylogenetic analysis of *Rickettsia* spp. based on the *gltA, ompA, ompB* and *sca4* genes revealed that the sequences obtained could be classified into two groups, representing two different rickettsial species, *R. slovaca* and *R. raoultii*. Previous studies reported that SFG rickettsiae including *R. aeschlimannii, R. conorii, R. sibirica, R. massiliae*, Candidatus *R. barbariae, R. raoultii*, and *R. slovaca*, were detected in field-collected *Ha. qinghaiensis, D. marginatus, D. abaensis, D. silvarum, Ixodes crenulatus, Rhipicephalus turanicus*, and *D. nuttalli* in northwestern China (23, 24). Consistent with our findings in this study, it is common to detect *R. raoultii* and *R. slovaca* in *D. nuttalli* ticks in previous studies (8, 23, 24). The two *Rickettsia* species were identified as pathogenic factors of human rickettsiosis in tick vectors and humans (39, 40, 61), and importantly, *R. raoultii* has been detected in humans in China (39, 40). These results suggest that humans and animals are susceptible to the pathogens due to exposure to *D. nuttalli* tick vectors in the sampling areas.

In conclusion, this study is the first to characterize the life cycle of collected *D. nuttalli* in the field from the Qinghai-Tibetan Plateau under laboratory conditions, and two species of SFG *Rickettsia* were identified in the midgut and salivary glands of males and females of both field-collected and first-laboratory generation adults. However, there are differences between laboratory conditions and natural conditions that impact tick survival and may influence the life cycle of laboratory-reared ticks. Moreover, the seasonal diapause of ticks must be considered because it may affect their life cycle and pathogen colonization under laboratory conditions. Our study provides new insights into the pathogen colonization of ticks on the Qinghai-Tibetan Plateau, and the relationships among hosts, ticks and pathogens.

## Data availability statement

The datasets presented in this study can be found in online repositories. The names of the repository/repositories and accession number(s) can be found in the article/supplementary material.

## Author contributions

YS: conceptualization, data curation, formal analysis, funding acquisition, resources, writing-original draft, and writing-review and editing. HM and JA: data curation, formal analysis, and investigation. MK: resources and writing-review and editing. JL: conceptualization, writing-original draft, and writing-review and editing. All authors contributed to the article and approved the submitted version.
